# The sequence preference of DNA methylation variation in mammalians

**DOI:** 10.1371/journal.pone.0186559

**Published:** 2017-10-18

**Authors:** Ling Zhang, Chan Gu, Lijiang Yang, Fuchou Tang, Yi Qin Gao

**Affiliations:** 1 Biodynamic Optical Imaging Center (BIOPIC), School of Life Sciences, Peking University, Beijing, China; 2 Institute of Theoretical and Computational Chemistry, College of Chemistry and Molecular Engineering, Peking University, Beijing, China; 3 Prenatal Diagnosis Center, Department of Obstetrics and Gynecology, Ministry of Education Key Laboratory of Obstetric, Gynecologic and Pediatric Diseases and Birth Defects, West China Second University Hospital, Sichuan University, Chengdu, Sichuan, China; Beijing Cancer Hospital, CHINA

## Abstract

Methylation of cytosine at the 5 position of the pyrimidine ring is the most prevalent and significant epigenetic modifications in mammalian DNA. The CpG methylation level shows a bimodal distribution but the bimodality can be overestimated due to the heterogeneity of per-base depth. Here, we developed an algorithm to eliminate the effect of per-base depth inhomogeneity on the bimodality and obtained a random CpG methylation distribution. By quantifying the deviation of the observed methylation distribution and the random one using the information formula, we find that in tetranucleotides 5’-N_5_CGN_3_-3’ (N_5_, N_3_ = A, C, G or T), GCGN_3_ and CCGN_3_ show less apparent deviation than ACGN_3_ and TCGN_3_, indicating that GCGN_3_ and CCGN_3_ are less variant in their level of methylation. The methylation variation of N_5_CGN_3_ are conserved among different cells, tissues and species, implying common features in the mechanisms of methylation and demethylation, presumably mediated by DNMTs and TETs in mammalians, respectively. Sequence dependence of DNA methylation variation also relates to gene regulatory and promotes the reexamination of the role of DNA sequence in fundamental biological processes.

## Introduction

Eukaryotic chromosomes carry genomic information of individual growth and development, which is stored not only in DNA sequence, but also in epigenetic information such as DNA methylation and histone modifications [1, 2]. Methylation of cytosine at the 5 position of the pyrimidine ring (5mC) is the most prevalent and significant epigenetic modification in mammalian DNA [3], which is generally associated with cellular processes such as cell differentiation, X chromosome inactivation, transposon silencing, genomic imprinting and tumourigenesis [4, 5]. About 1.5 percent of the cytosines in mammalian genomes are methylated [6]. The vast majority of 5mC exists at CpG dinucleotides, and more than 60 percent of CpG dinucleotides are methylated in mammalian genome [7]. One interesting phenomenon is that the distribution of methylcytosines in the genome of many species and cell types is not random [8], and the CpG methylation levels exhibit a bimodal distribution indicating a sequence/site specificity. The measured CpG methylation levels distribution peaking at 0 and 1 [9, 10], which implies that during sequencing a large population of the CpG sites remain either unmethylated or fully methylated. Most of the CpG dinucleotides are hypermethylated, whereas those in CpG islands keep hypomethylated in adult cells [11]. One of the possible functions of this bimodal pattern is to keep the factor-mediated basal transcription profile of the preimplantation embryo [12].

The development of the next generation sequencing (NGS) technology has made the methylome of many species available. Lister *et al*. performed the first genome-wide single-base resolution sequencing of *Arabidopsis* methylome in 2008 [13]. followed by the methylome of h1 human embryonic stem cells (ESCs) and human induced pluripotent stem cells (iPSCs) [14]. In 2013 the same group implemented the sequencing of DNA methylation in the frontal cortex of human and mice covering their life span [15]. Contemporaneously, many other researchers also obtained methylomes of other tissues and species. For example, Guo *et al*. carried out the sequencing of human early embryos and human primordial germ cells (PGCs) [16], and another important progress is that the epigenomes of 18 tissue types from 4 individuals of high coverage were obtained by Schultz *et al*. [17]. 111 reference human epigenomes were obtained by the NIH Roadmap Epigenomics Consortium in 2015 [18]. The abundant methylome data from previous work enable us to exploit the bioinformatics approach for a better understanding of DNA methylation.

Despite the modern experimental sequencing technologies have achieved significant success, researchers are still confronted with great challenges. For example, the non-uniform sequencing depth of each CpG can lead to the overestimation of the bimodality of CpG methylation. We use sequencing depth to denote the coverage of a CpG dinucleotide in sequencing. In this paper we eliminated the bias of the bimodality of CpG methylation caused by the heterogeneous sequencing depths to the bimodality of CpG methylation and analyzed the bimodal distributions of CpG methylation for a large variety of mammalian cell and tissue types. Our goal in this study is to investigate whether the flanking bases have an effect on the CpG methylation and whether such an effect, if exists, could shed light on the mechanism of methylation and demethylation. Interestingly, these analyses did reveal a sequence dependent feature that is common for all samples analyzed. In tetranucleotides 5’-N_5_CGN_3_-3’ (N_5_, N_3_ = A, C, G or T), the GCGN_3_ and CCGN_3_ show a lower tendency of methylation variation, compared to ACGN_3_ and TCGN_3_. Molecular dynamics simulations were then used to understand the origin of such differences. The intrinsic DNA structure parameters of CpG/5mCpG sites are found to be significantly affected by the flanking bases N_5_ and N_3_. The structural differences between different sequences provide a possible explanation of the variation of methylation properties and further may clarify the gene regulatory mechanisms.

## Materials and methods

### Data resources

Methylomes of mouse and human brain cells are from Lister *et al*. [15]. Human embryonic stem cells (ESCs) and human induced pluripotent stem cells (iPSCs) are taken from Lister *et al*. [14], and methylomes of human normal somatic cells are from Schultz *et al*. [17]. Methylomes of human primordial germ cells (PGCs) and the neighboring somatic cells (gonadal somatic cells, SOMAs) are taken from Guo *et al*. [16]. The details of each sample are listed in S1–S6 Tables. We categorized the data into six groups: mouse brain cells, human brain cells, human ESCs and iPSCs, human normal somatic cells, human PGCs and human SOMAs.

### Analysis of methylation level

Following the literature, we use *β*_*i*_ to represent the measured methylation level of the *i*th CpG site.

$$\beta_{i}=\frac{M_{i}}{T_{i}}$$ (1)

where *T*_*i*_ is the sequencing depth of the *i*th CpG site and *M*_*i*_ is its measured methylation frequency. Accordingly, one can obtain the observed probability distribution of *β* through a simple count of the appearing frequency, which is normally represented by a discretized function *r*_*obs*_(*β*),

$$r_{obs}(\beta )=\frac{\sum_{i}\delta (\beta -\beta_{i})}{N}$$ (2)

Where *N* is the total number of all CpG sites and δ(∙) is the delta function.

If all the sequencing depth *T* is large enough, one can get the accurate methylation level *β* of each CpG site as well as the according *β* distribution (denoted as *r*_*acc*_(*β*)). However, due to the limited and non-uniform sequencing depth in the experiments, one gets only a measured methylation level of each CpG site (*β*_*i*_) (Eq 1) and its distribution *r*_*obs*_(*β*) (Eq 2).

In Eq 2, both the denominator and nominator are integers, therefore *r*_*obs*_(*β*) can only come out among a group of fraction numbers, that means the continuous *r*_*acc*_(*β*) is “discretized” as *r*_*obs*_(*β*). Due to the non-uniform sequencing depth, *r*_*obs*_(0) and *r*_*obs*_(1) can be overestimated compared to the *r*_*acc*_(0) and *r*_*acc*_(1), respectively. For a specific sequencing depth *T*, *β* can only come out as $0,\frac{1}{T},\frac{2}{T},\ldots ,1$, but not any other values. For example, a sequencing depth of 2 yields possible methylation levels of 0, ½, and 1, while a depth of 4 yields 0, ¼, ½, ¾, and 1. If one directly counts the observed values, there would be a bias towards more favored values of 0, ½, and 1 compared to ¼ and ¾. On the other hand, every sequencing depth can generate a *β* values of 0 and 1, thus *β* values of 0 and 1 appear more frequently than other values in a simple counting strategy, which will result in an artifact in the observed bimodal distribution, especially when a bunch of low sequencing depth CpG sites are included in the analyses.

To eliminate the bias caused by the non-uniform sequencing depth, we computationally generate an artificial sample in which the sequencing depth of each CpG site is identical to the experimental value but the methylation frequency of each CpG site obeys a binomial distribution, which means that the methylation level of each CpG site is random. The methylation distribution of the randomized sample is denoted as *r*_*ran*_(*β*). The difference between the direct observation (*r*_*obs*_(*β*)) and the random counterpart (*r*_*ran*_(*β*)) is free of sequencing depth bias and it characterizes how much the observed methylation level distribution deviates from the random distribution.

In the randomized sample, for the *i*th CpG site with a sequencing depth *T*_*i*_, the binomial distribution [19] of the methylation frequency *n*(*n* = 0,1…*T*_*i*_) can be formulated as,

$$P(T_{i},n)={∁}_{T_{i}}^{n}p^{n}{\left(1-p\right)}^{T_{i}-n}$$ (3)

where *p* is defined as the average of the observed methylation level of all CpG sites (Eq 4).

$$p=\frac{\sum_{i=1}^{N}\beta_{i}}{N}$$ (4)

The probability distribution of *β* in the random case for the methylome can then be calculated by Eq 5

$$r_{ran}(\beta )=\frac{\sum_{i}\sum_{n}P(T_{i},n)\delta (n/T_{i}-\beta )}{N}$$ (5)

To quantify the deviation of the observed methylation distribution (*r*_*obs*_(*β*)) and that in the random case (*r*_*ran*_(*β*)), one can use the direct difference of them (*d*(*β*) = *r*_*obs*_(*β*) − *r*_*ran*_(*β*)) or another choice is the relative entropy (also known as Kullback-Leibler divergence) [20]. In the latter approach, larger relative entropy indicates a stronger deviation between the two functions. The deviation of the observed distribution from the random distribution is expressed in Eq 6.

$$KL(\beta )=KL(r_{obs}(\beta )||r_{ran}(\beta ))=\sum_{\beta }r_{obs}(\beta )\log_{2}\frac{r_{obs}(\beta )}{r_{ran}(\beta )}$$ (6)

Specifically, for *KL*(0) and *KL*(1), we have

$$KL(0)=KL(r_{obs}(0)||r_{ran}(0))=r_{obs}(0)\log_{2}\frac{r_{obs}(0)}{r_{ran}(0)}$$ (7)

$$KL(1)=KL(r_{obs}(1)||r_{ran}(1))=r_{obs}(1)\log_{2}\frac{r_{obs}(1)}{r_{ran}(1)}$$ (8)

The biological implication of the deviation between the observed methylation level distribution and the random distribution at 0 (*d*(0) or *KL*(0)) and 1 (*d*(1) or *KL*(1)) are that they reflect the methylation and demethylation variation (or conservation conversely), respectively. As in the normal bisulfite sequencing, the mixed cell populations were sequenced and the measured methylation level *β*_*i*_ for the *i*th CpG site approximately indicates the ratio of the reads with the *i*th CpG site methylated to the total reads with *i*th CpG site detected, the greater difference between the observed methylation level distribution and the random distribution in the 0 (*d*(0) or *KL*(0)) means the CpG is prone to keep its unmethylated state.

For tetranucleotide N_5_CGN_3_ (N_5_, N_3_ = A, C, G or T), there are 16 possible different sequences with the two DNA chains considered separately. We calculate all the 16 *KL*(*β*) to investigate the influence of the flanking sequences to the CpG sites. The *p* in Eq 3 is thus the average methylation level of the corresponding tetranucleotide (ACGA, CCGT *et al*.).

## Results

### The bimodal distribution of CpG methylation level and methylation variation

**Fig 1:**
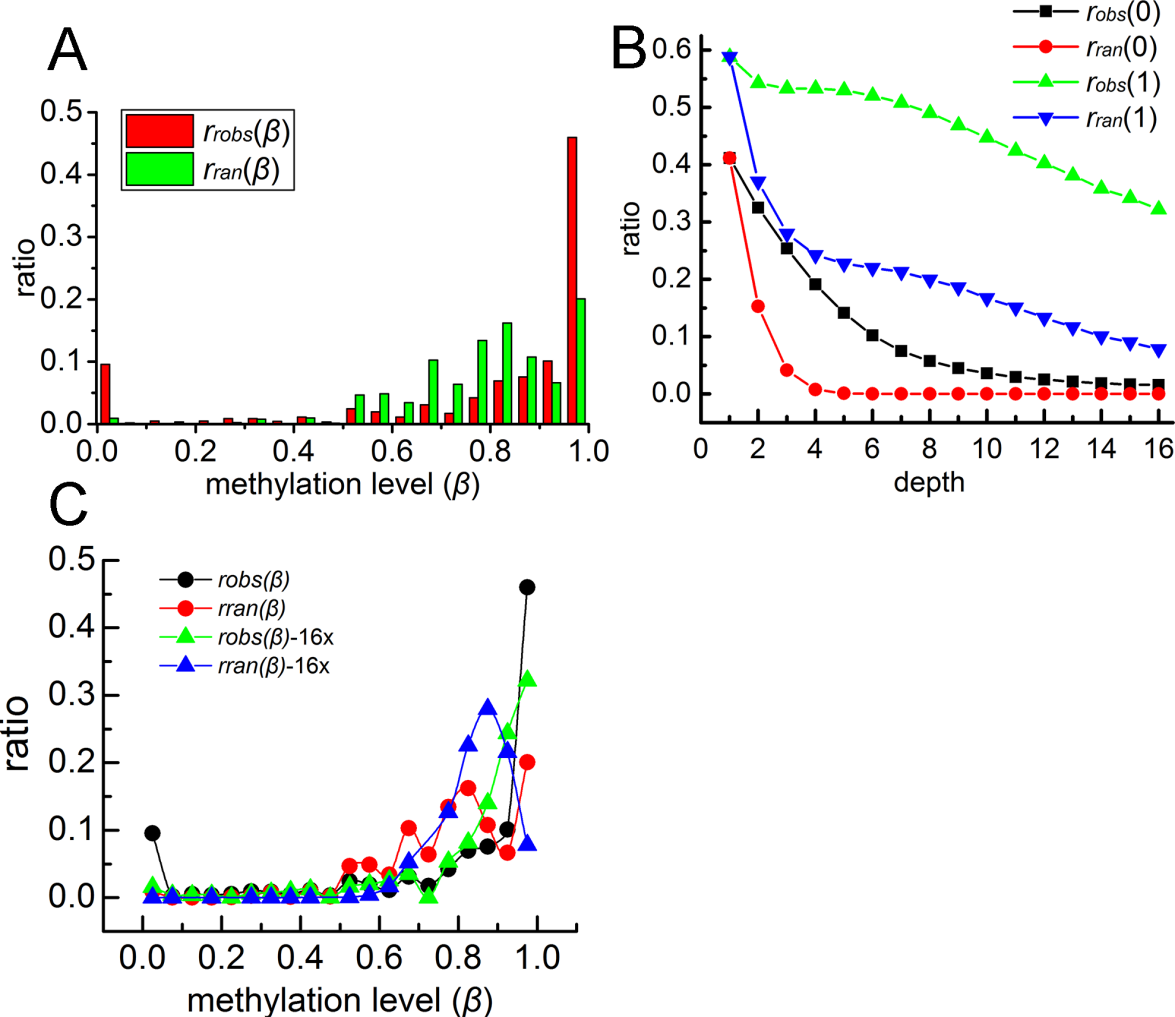
The bimodal distribution of CpG methylation in human brain samples. **(A)** The bimodal bias caused by the inhomogeneity of sequencing depth in human brain cells (chromosome 1 of the 12yr sample). **(B)** The observed ratio and the random ratio of 0 and 1 end of the methylation level distribution in different sequencing depths. **(C)** The observed and random methylation level distribution of all sequencing depth (black circle and red circle respectively) and the observed and random methylation level distribution under the sequencing depth of 16x is in green triangle and blue triangle respectively.

As reported earlier, the distribution of the mammalian CpG methylation level is bimodal, with peaks seen at *β* = 0 and *β* = 1. We calculated the CpG methylation level distribution for all six categories of samples (66 samples in total, Materials and methods). The observed methylation level distribution does show a typical bimodal pattern (Fig 1A, red). For comparison, we also show in this figure the randomized methylation level following the binomial distribution with the experimental sequence depth taken into account (Materials and methods, Eq 5, Fig 1A, green). The randomized methylation level distribution also exposits the bimodal feature, which shows that even if a simple binomial distribution assumption with uneven sequencing depth can result in bimodality, although appears to a less extent than the experimental observation. The more pronounced peaks at *β* = 0 and *β* = 1 in the experimental data (Fig 1A, red) indicates that the methylation level is indeed biased toward 0 or 1, even after the removal of the bias caused by the sequencing depth.

To illuminate the effect of sequencing depth on the observed bimodality of CpG methylation level distribution, the *r*_*obs*_(0), *r*_*ran*_(0), *r*_*obs*_(1) and *r*_*ran*_(1) of different sequencing depth is shown in Fig 1B, which clearly shows that with the increase of sequencing depth, the bimodality of methylation level become less obvious. We also show in the Supporting information the observed and random distribution of CpG methylation in each sequencing depth (S1 Fig). It can be seen that the non-uniform sequencing depth does lead to an overestimated distribution at various *β* values, especially at *β* = 0 and *β* = 1, even though a binomial distribution is assumed. Therefore, it is important to eliminate the disadvantage of uneven sequencing depth. For comparison, we show that the methylation level distribution is not biased toward for *β* = 0 or *β* = 1 when a uniform sequencing depth is used for each CpG site, as seen in Fig 1C.

### The methylation variation of tetranucleotides in human brain cells

The average methylation levels of N_5_CGN_3_ (N_5_, N_3_ = A, C, G or T) in chromosome 1 of brain samples are shown in S2 Fig. For N_5_CGC (N_5_ = A, C, G or T), the average methylation levels of ACGC and TCGC are higher than that of GCGC and CCGC. This ranking order remains for N_5_CGG (N_5_ = A, C, G or T) while N_5_CGA and N_5_CGT (N_5_ = A, C, G or T) have different average methylation level ranking orders. Therefore, the average methylation level of N_5_CGN_3_ (N_5_, N_3_ = A, C, G or T) does not show a consensus dependence on the flanking bases.

**Fig 2:**
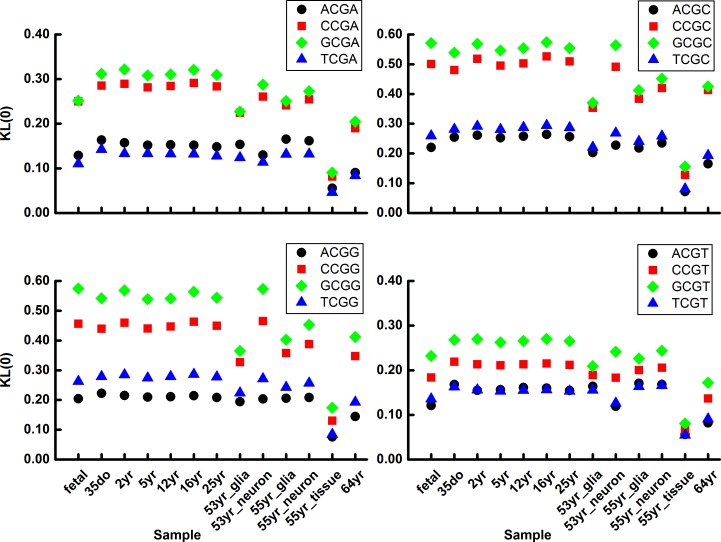
Methylation variation of N_5_CGA (upper left), N_5_CGC (upper right), N_5_CGG (lower left) and N_5_CGT (lower right) of chromosome 1 in human brain cells. The *KL*(0) of ACGN_3_, CCGN_3_, GCGN_3_ and TCGN_3_ are represented as black circle, red square, green diamond and blue triangle, respectively. N_5_, N_3_ = A, C, G or T.

**Fig 3:**
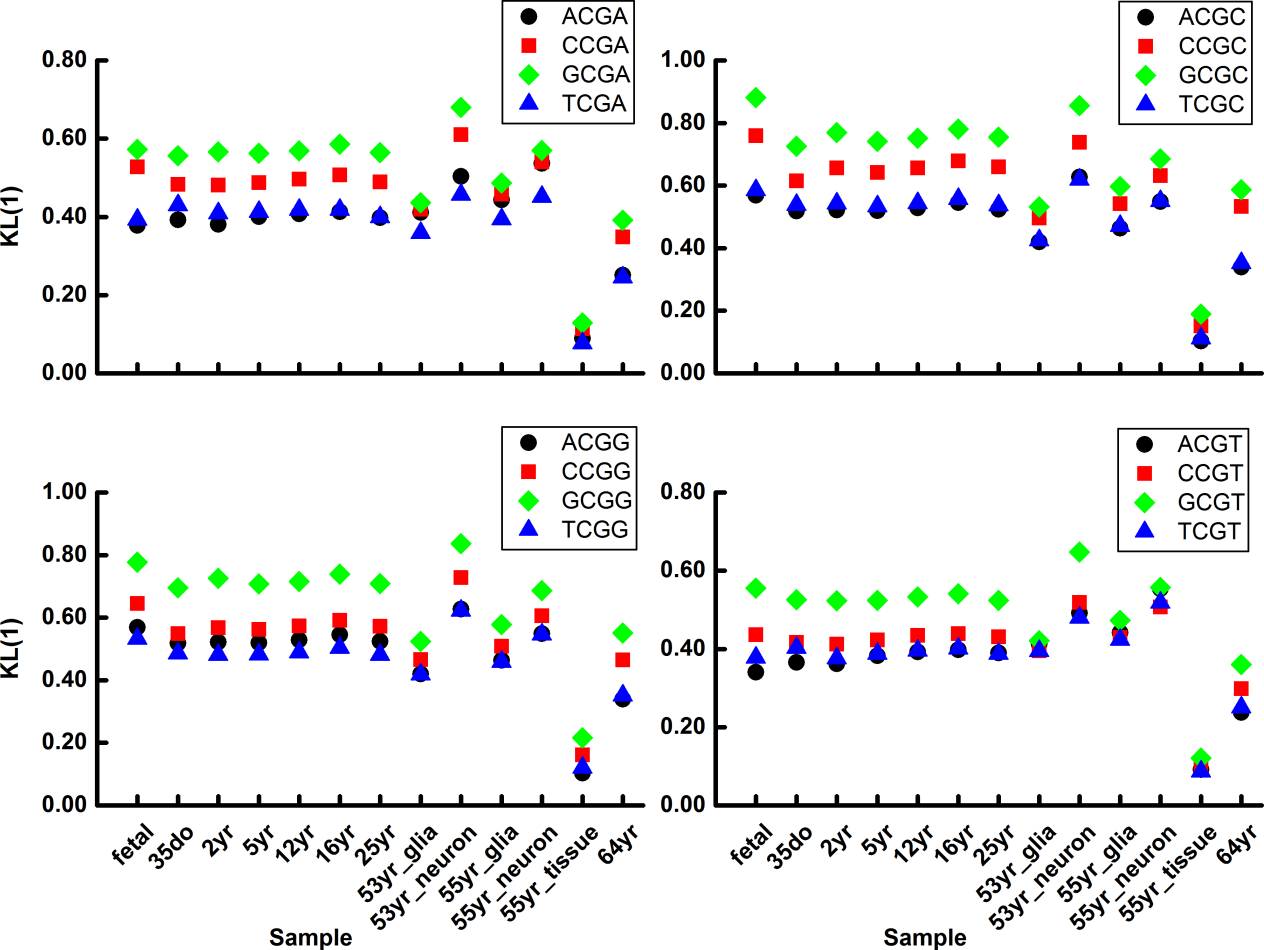
Demethylation variation of N_5_CGA (upper left), N_5_CGC (upper right), N_5_CGG (lower left) and (D) N_5_CGT (lower right) in human brain cells.

**Fig 4:**
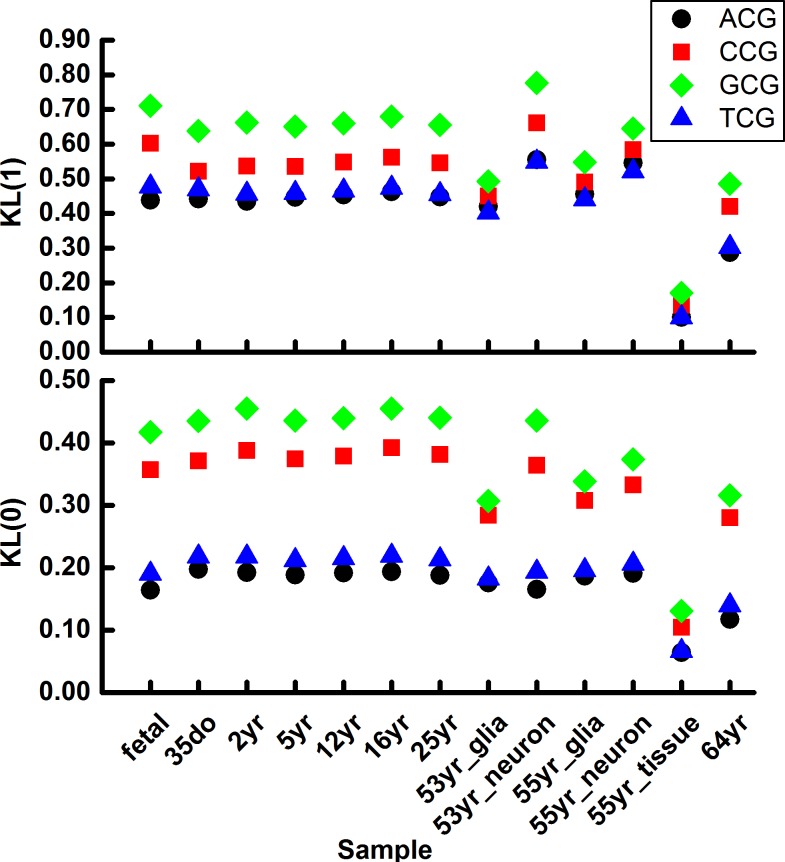
Methylation variation (above) and demethylation variation (below) of trinucleotide N_5_CG of chromosome 1 in human brain samples.

We then performed a detailed analysis of *KL*(0) and *KL*(1) (Materials and methods, Eqs 7 and 8) to investigate how the methylation/demethylation pattern of a particular CpG site is conserved and whether a simple flanking sequence dependence can be identified. We characterized the difference between observed and randomized methylation level distribution functions for the 16 tetranucleotides in chromosome 1 of human brain samples using the relative entropy approach. The calculated results (Figs 2 and 3) exhibit a clear trend that both *KL*(0) and *KL*(1) of GCGN_3_ and CCGN_3_ is greater than that of TCGN_3_ and ACGN_3_, independent of the N_3_ base, indicating that the CpG methylation of GCGN_3_ and CCGN_3_ is more conserved than that of ACGN_3_ or TCGN_3_. The results also make clear that the 5’ but not 3’ base of the CpG affects the methylation variation of the CpG in a consensus way (Fig 4). In the following text, we focus on the N_5_ base and examine the methylation in the N_5_CG trinucleotides. For comparison, we also calculated the direct deviation of *r*_*obs*_(*β*) and *r*_*ran*_(*β*), which is denoted as *d*(*β*) (*β* = 0, 1) (S3 Fig). The *d*(0) and *d*(1) show a similar trend as *KL*(0) and *KL*(1).

In addition, we calculated the *KL*(0) and *KL*(1) of trinucleotide N_5_CG of other 23 chromosomes in human brain samples. The results (S4 and S5 Figs) shows that the pattern is conserved among all of the 22 autosomes and the 2 allosomes, indicating that the pattern is not chromosome specific.

### The methylation and demethylation variation of trinucleotides are conserved among different tissues and species

**Fig 5:**
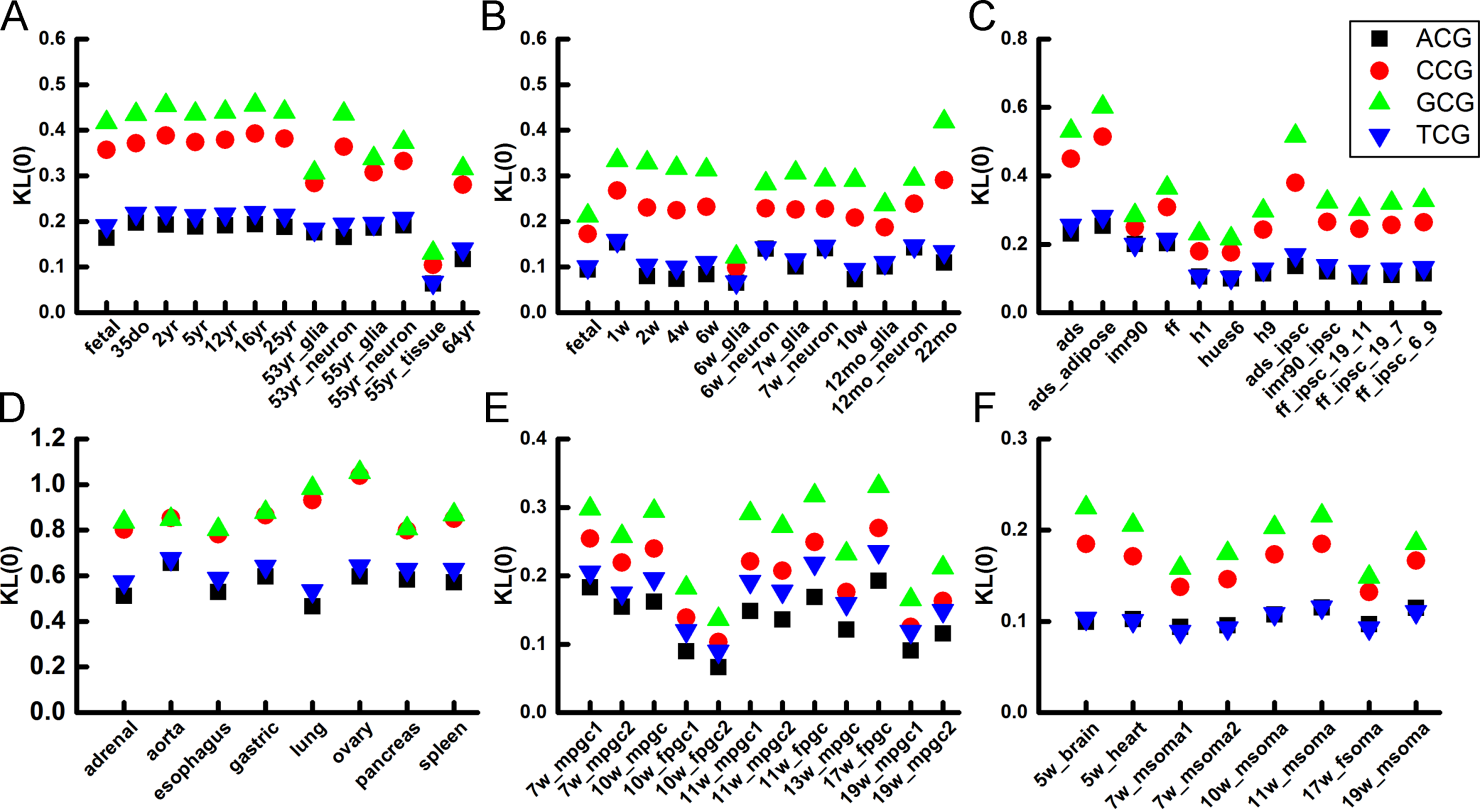
Methylation variation of N_5_CG in human and mouse cells. **(A)** Human brain samples. **(B)** Mouse brain cells. **(C)** Human ESCs and iPSCs. **(D)** Human normal somatic cells. **(E)** Human PGCs. **(F)** Human gonadal somatic cells (SOMAs).

**Fig 6:**
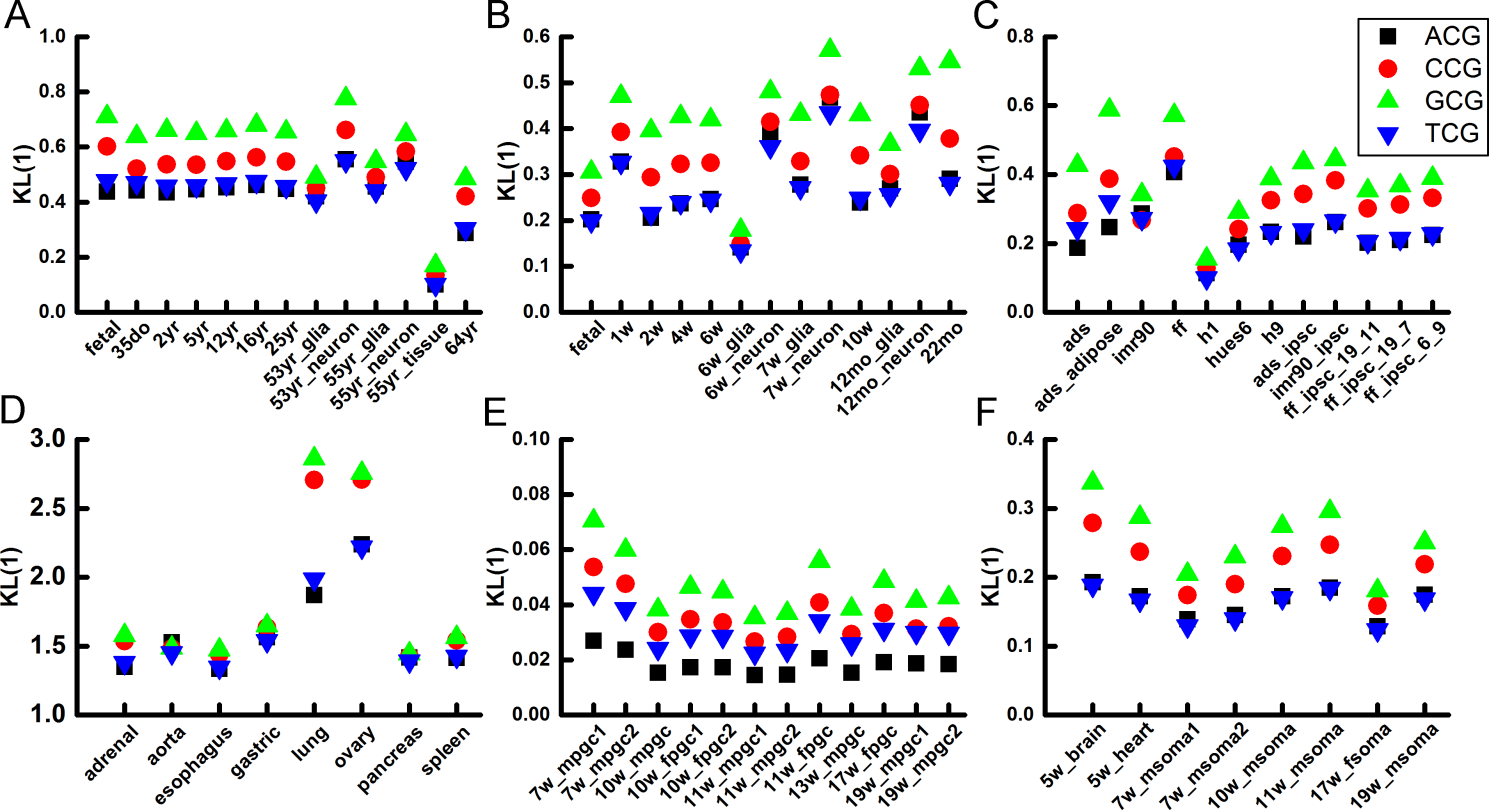
Demethylation variation of N_5_CG in human and mouse cells. **(A)** Human brain samples. **(B)** Mouse brain cells. **(C)** Human ESCs and iPSCs. **(D)**Human normal somatic cells. **(E)** Human PGCs. **(F)** Human gonadal somatic cells (SOMAs).

As trinucleotides in human brain samples show a conserved pattern that TCG and ACG are more variable in methylation level than GCG and CCG for all chromosomes, we wonder if this pattern is conserved in other tissues and species. Firstly, in 8 human somatic cells (including lung, gastric, spleen *et al*.), the *KL*(0) and *KL*(1) of GCG and CCG is also greater than that of TCG and ACG, indicating lower methylation variation of GCG and CCG. Next, in human ESCs and iPSCs the trend of *KL*(0) and *KL*(1) that GCG, CCG > ACG, TCG is also conserved. Next we investigate whether the methylation variation is conserved during early embryonic development and analyzed samples from human PGCs and SOMAs. Human PGCs are hypomethylated due to the large scale of demethylation during the embryogenesis. 12 different stages or gender of human PGCs and 8 SOMAs were analyzed. Although the methylation levels of these human samples vary dramatically, we found that all *KL*(0) and *KL*(1) values follow the same order of GCG, CCG > TCG, ACG. Finally, to examine whether the simple trend found in these analyses persists across different mammalian species, we also analyzed methylome data of the mouse brain cells. The *KL*(0) and *KL*(1) of all analyzed samples are shown in Figs 5 and 6. The ranking order, GCG, CCG > TCG, ACG, for *KL*(0) and *KL*(1) are strictly followed by these cells.

In summary, the sequence dependence of CpG methylation variability is conserved across different tissues, developmental stages, ages, genders and species, which indicates that the variations in methylation and demethylation of GCG and CCG are lower than that of ACG and TCG.

### The methylation variation in partially methylated domains and the effect of sequencing depth on methylation variation

**Fig 7:**
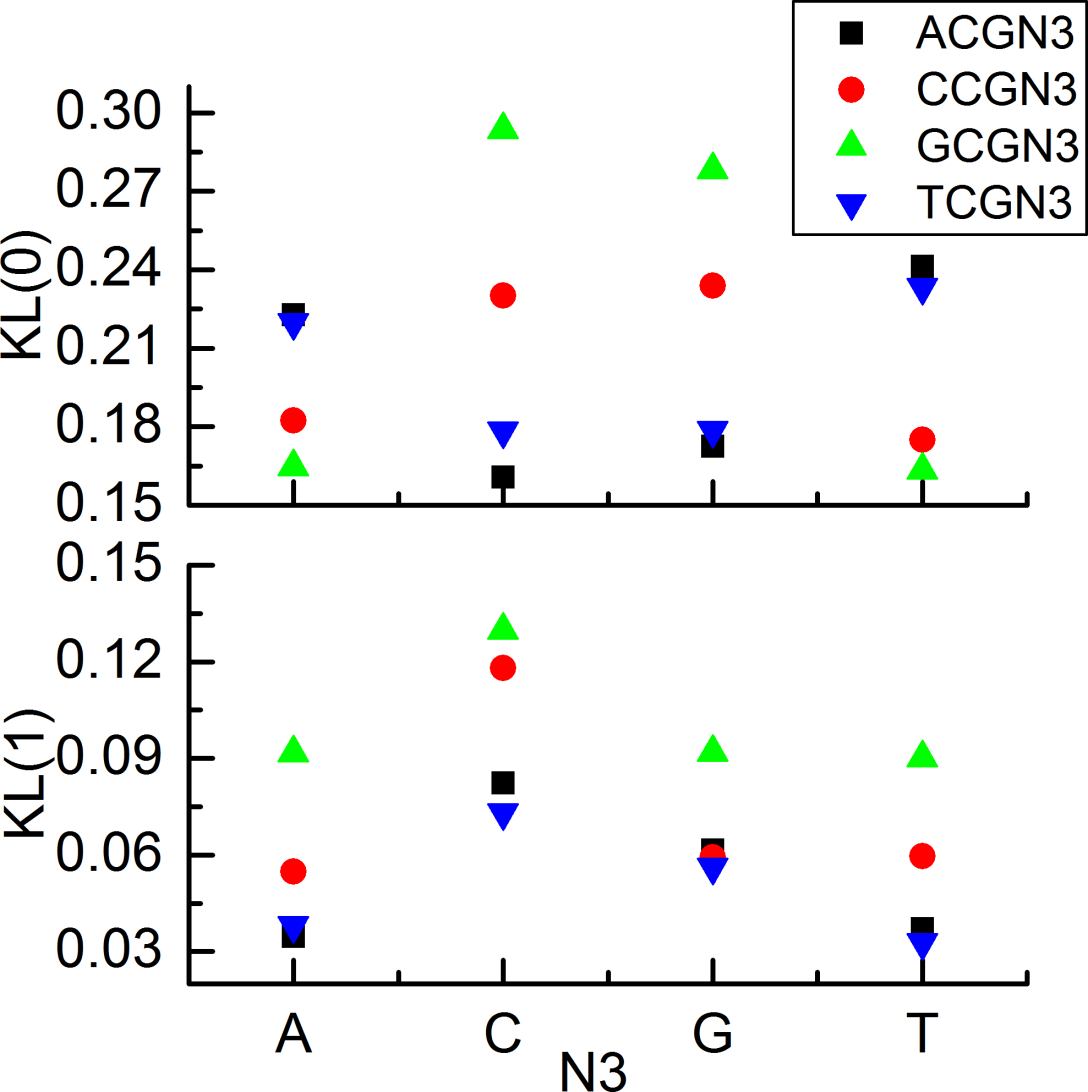
Methylation variation (above) and demethylation variation (below) in PMDs (chr1 of IMR90 cell lines).

Partially methylated domains (PMDs) are the hypomethylated regions in specific cells such as IMR90 cell lines, human placenta and certain cancer cells and gene expression in PMDs are repressed [6, 21, 22]. We calculated the *KL*(0) and *KL*(1) of PMDs in the IMR90 cell lines (Fig 7) (PMD regions are from Lister *et al*. [6].). For *KL*(1), the methylation variation of GCG and CCG is smaller than that of ACG and TCG, consistent with the results obtained for the whole chromosome. However, the *KL*(0) of N_5_CGA and N_5_CGT shows an opposite trend. These orders of methylation variability order are conserved among all 23 chromosomes of IMR90 cell lines (S6 and S7 Figs). We speculate that the higher methylation variability (*KL*(0)) of GCGN_3_ and CCGN_3_ (N_3_ = A,T) in PMDs may reflect that these tetranucleotides are selectively unmethylated (S8 Fig).

**Fig 8:**
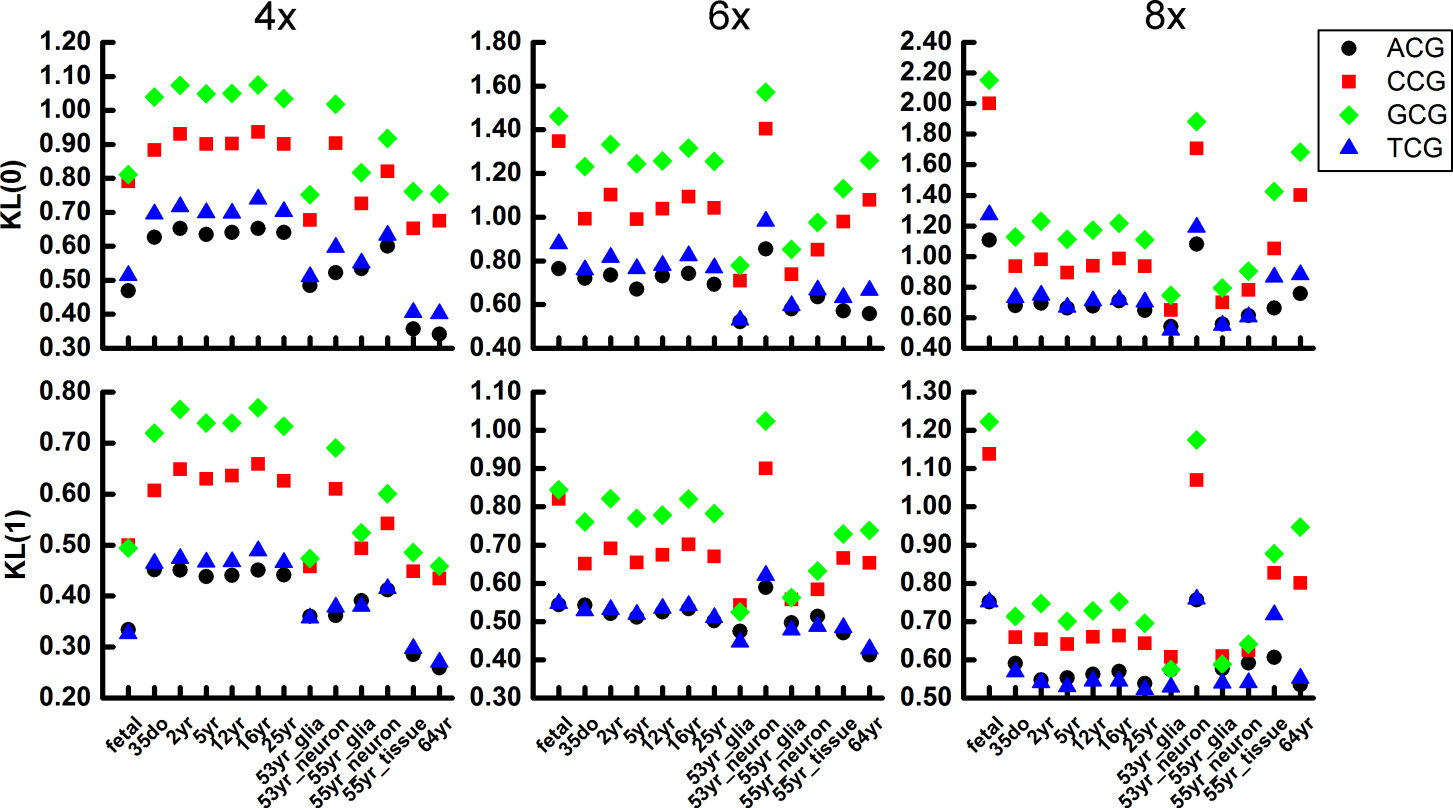
Methylation variation (above) and demethylation variation (below) in human brain cells of difference sequencing depth.

To further investigate the effect of sequencing depth to the methylation and demethylation variation, we calculated the methylation and demethylation variation of different sequencing depth. In Fig 8, we show the *KL*(0) and *KL*(1) of the sequencing depth 4x, 6x and 8x. The *KL*(0) and *KL*(1) increases with the larger sequencing depth, but the methylation and demethylation trend GCG, CCG > TCG, ACG is also conserved.

## Discussion

The current study shows that the variability of DNA methylation level of a particular CpG site is noticeably affected by its flanking bases. In particular, since the methylation and demethylation processes are mediated by homologous DNMTs [23, 24] and TETs [25, 26] in both human and mouse, the identical ranking order of variability may indicate similar molecular mechanisms used by the two different species. It is therefore interesting to look into how methylation and demethylation are affected by the sequence-dependent intrinsic DNA structural properties. We therefore performed MD simulations to examine how flanking bases affect the local structural properties of the CpG step (S1 Text).

We first note that a number of crystal structures of DNMTs and TETs have been reported and helped illuminate the molecular mechanisms of methylation and demethylation in mammalians [23–25, 27–29]. Both DNMTs and TETs make use of the base flipping mechanism in order to modify the target cytosine/methylcytiosine. Several steps involve in the base flipping mechanism: first, the target sites are recognized by the recognition domain of protein; the target cytosine or methylcytosine flips out via their interactions with the catalytic domain of the protein; and finally the target is modified in the active pocket[30–32]. Since the substrates of modification are cytosine for methylation and methylcytosine for demethylation, the chemical reactions of methylation (or demethylation) in the catalytic pocket are the same among N_5_CGN_3_ (or N_5_mCGN_3_), leaving the base flipping process a possible cause of the flanking base dependence. Since the target cytosine (or methylcytosine) mainly interacts with the DNMTs (or TETs) from the DNA minor groove[23, 29], it is expected that an accessible minor groove would facilitate the base flipping. In the B form DNA structure, the atoms of cytosine/methylcytosine can be classified as the “minor groove atoms” or “major groove atoms” based on the groove they face. We compared the probability distributions of Solvent Accessible Surface Area (SASA) of cytosine in N_5_CGN_3_ (S9 Fig) and methylcytosine in N_5_mCGN_3_ (S10 Fig) of the different DNA sequences. The SASA describes the surface area of a molecule which is accessible to the solvent. The SASA in this paper were calculated using the Naccess program (http://www.bioinf.man.ac.uk/naccess/). As seen in S6 and S7 Figs, the average SASA of minor groove atoms in GXGN_3_ and CXGN_3_ (X = C or mC) are in general smaller than those in AXGN_3_ and TXGN_3_ (X = C or mC) for both cytosine or the methylcytosine, indicating that the target cytosine/methylcytosine in the former two types of tetranucleotides are less accessible to the proteins, and thus a less favored DNA/protein interaction, than that in the latter two. The SASA distributions of O2 atom of C/5mC are shown in S9 and S10 Figs, respectively. It can be clearly seen from these figures that AXGN_3_ and TXGN_3_ are characterized by smaller O2 SASA values than GXGN_3_ and CXGN_3_. These results are consistent with AXGN_3_ and TXGN_3_ being more prone to form pre-flipping states than GXGN_3_ and CXGN_3_. Generally, the most stable hydrogen bond is embedded inside the base pair, and the least stable one is exposed to the major groove. By comparing the stabilities of hydrogen bonds between O2 of C/5mC-N2 of G, which directly contacts with the active-site loop, we found that the formation probability of hydrogen bonds between O2 of C/5mC-N2 of G for GXGN_3_ and CXGN_3_ are typically lower than that of AXGN_3_ and TXGN_3_, suggesting that the hydrogen bonds O2 of C/5mC-N2 of G in AXGN_3_ and TXGN_3_ are normally less stable than that in GXGN_3_ and CXGN_3_. Such a result is again in accordance with the higher variation of AXGN_3_ and TXGN_3_ than GXGN_3_ and CXGN_3_.

DNMT3A/3B is anchored to chromatins through the co-factor DNMT3L, and TETs interact with chromatins by the CXXC domain at the N terminal. DNA wrapped on histones adopts bending conformations, implying that bending may be beneficial to base flipping, which is also suggested by the bending DNA conformations in the ngTET1-DNA and hTET2-DNA crystal structures [25, 26] as well as the two DNA segments from the HhaI-DNA complex superimposed onto the DNMT3A-DNMT3L tetramer [23, 33]. The bending magnitude is usually adopted to describe the degree of DNA bending, which is calculated with roll and tilt angles [34]. We calculated the distributions of bending magnitudes around the target CpG/5mCpG sites in N_5_XGN_3_ (S11 and S12 Figs), with roll and tilt angles obtained by 3DNA program [35] and found that in general AXGN_3_ and TXGN_3_ have higher average bending magnitudes than GXGN_3_ and CXGN_3_.

These results all indicate that compared to GXGN_3_ and CXGN_3_, AXGN_3_ and TXGN_3_ adopt conformations that are likely more susceptible to interact with the environment, consistent with the latter two having higher methylation and demethylation variability. In summary, the structural properties of different sequences are consistent with their differences on the bimodality of methylation levels on the genome scale, providing a possible explanation to our sequencing data analysis.

Besides the relation to the structural properties, the methylation variation among different tetranucleotides also provides possible functional implications. Gene regulation is one of the most fundamental issues in understanding biological process such as cell differentiation, tumorigenesis and embryonic development, and recent studies provided important hints on the relationship between DNA methylation and gene expression [18]. The most prevalent paradigm of the regulation of gene expression levels through DNA methylation is that the hypermethylation of gene promoters or CpG islands relates to the repression of gene expression. But recent research reveals that methylation of distal regulatory sites (such as enhancers) also affects the gene expression, especially in transformed cells [36, 37]. Our result indicates that with CCG and GCG the methylation level is better maintained, which is expected to be important for the maintenance of biological properties including gene expression level. Accordingly, the promotors, the methylation of which affects significantly gene expression, is richer in CCG/GCG than ACG/TCG. For example, in promoters of chromosome 1 in the 12yr brain sample, the numbers of CCG and GCG trinucleotides are 63242 and 60158, respectively, whereas those of ACG and TCG are 32034 and 32294, respectively. Meanwhile, the average methylation level of CCG (0.263) and GCG (0.258) are also significantly lower than those of ACG (0.37) and TCG (0.359), suggesting their differences in regulating gene expression through methylation. As the methylation state of promoters in a specific cell type is relatively stable, the higher abundance and lower methylation level of CCG and GCG in promoter regions agree with its relatively stable methylation state.

Nevertheless, the maintenance of DNA methylation pattern relies on the dynamic balance between methylation and demethylation and other processes, and gene expression is a complex event involving hierarchical regulatory mechanisms. For example, the sequence of genes and their regulatory elements such as promoters and enhancers, the epigenetic modifications including DNA methylation and histone modifications, and the three-dimentional chromatin structure can all regulate the temporal and spatial specific expression of genes. We believe that DNA sequence is the infrastructure of gene regulatory and the different methylation variation of tetranucleotides N_5_CGN_3_ illuminated in this work may provide further useful information in the relation among DNA sequence and structure, DNA methylation and gene expression regulation.

## Conclusions

We show in this study that the variability of the CpG methylation level is significantly affected by the bases flanking the CpG base step. We analyzed the CpG methylation level distribution and especially the observed bimodality. For tetranucleotides N_5_CGN_3_, the methylation variation of GCGN_3_ and CCGN_3_ are less pronounced than that of ACGN_3_ and TCGN_3_, suggesting GCGN_3_ and CCGN_3_ tend to be more conserved in cytosine methylation and demethylation. This flanking base dependence of CpG methylation variability is conserved among different cells, tissues and species, strongly suggesting a common mechanism of methylation and demethylation, which are mediated by DNMTs and TETs in mammalian, respectively. In summary, a quantitative description of the bimodal methylation level distribution and its sequence dependence were provided by the analyses of a large amount of methylomes, which provides implications to connect the sequence dependent methylation conservation and the DNA local structure.

## Supporting information

S1 Text Simulation details of molecular dynamics. (PDF)

S1 Fig The observed and random distribution of CpG methylation in different sequencing depth. (PDF)

S2 Fig Average methylation level of N_5_CGA, N_5_CGC, N_5_CGG and N_5_CGT in human brain cells. (PDF)

S3 Fig Comparison between the *d*(0), *KL*(0) and *d*(1), *KL*(1) in the chromosome 1 of human brain samples. (PDF)

S4 Fig The methylation variation of all chromosomes in human brain samples. (PDF)

S5 Fig The demethylation variation of trinucleotides in all chromosomes in human brain samples. (PDF)

S6 Fig The methylation variation of tetranucleotide in PMDs of all chromosomes in IMR90 cell line. (PDF)

S7 Fig The demethylation variation of tetranucleotide in PMDs of all chromosomes in IMR90 cell line. (PDF)

S8 Fig The average methylation level of the tetranucleotides of chromosome 1 in IMR90 cell lines. (PDF)

S9 Fig The distributions of SASA of O2 for (A) N_5_CGA, (B) N_5_CGC, (C) N_5_CGG and (D) N_5_CGT.
(PDF)

S10 Fig The distributions of SASA of O2 for (A) N_5_mCGA, (B) N_5_mCGC, (C) N_5_mGG and (D) N_5_mCGT.
(PDF)

S11 Fig The distribution of bending magnitudes of CpG sites relate to (A) N_5_CGA, (B) N_5_CGC, (C) N_5_CGG and (D) N_5_CGT.
(PDF)

S12 Fig The distribution of bending magnitudes of 5mCpG sites relate to (A) N_5_mCGA, (B) N_5_mCGC, (C) N_5_5mCGG and (D) N_5_mCGT.
(PDF)

S1 Table The detailed information of human brain samples. (PDF)

S2 Table The detailed information of mouse brain samples. (PDF)

S3 Table The detailed information of human ESCs and iPSCs samples. (PDF)

S4 Table The detailed information of human somatic cell samples. (PDF)

S5 Table The detailed information of human PGC samples. (PDF)

S6 Table The detailed information of human gonadal somatic (SOMA) samples. (PDF)
