# Supplementary material for: The sequence preference of DNA methylation variation in mammalians
Source: PLoS One. 2017 Oct 18;12(10):e0186559. doi: 10.1371/journal.pone.0186559 (PMC5646869; doi:10.1371/journal.pone.0186559)
Supplement: S1 Text — (PDF) [file pone.0186559.s001.pdf]

Supporting information

The sequence preference of DNA methylation variation in mammalians

Ling Zhang[1,2], Chan Gu[2,3], Lijiang Yang[1,2], Fuchou Tang[1], Yi Qin Gao[1,2,*]

[1] Biodynamic Optical Imaging Center (BIOPIC), School of Life Sciences, Peking University, Beijing, China

[2] Institute of Theoretical and Computational Chemistry, College of Chemistry and Molecular Engineering, Peking University, Beijing, China

[3] Prenatal Diagnosis Center, Department of Obstetrics and Gynecology, Ministry of Education Key Laboratory of Obstetric, Gynecologic and Pediatric Diseases and Birth Defects, West China Second University Hospital, Sichuan University, Chengdu, Sichuan, China

*Corresponding author
Email: gaoyq@pku.edu.cn (YQG)

Supporting method

Simulation details of molecular dynamics

As discussed earlier, one main purpose of this paper is to examine whether the methylome sequencing data reflect intrinsic DNA structural properties. Therefore, we performed MD simulations to search for the possible relationship between the differences of methylation levels among N_5XGN_3 (X=C or 5mC) found in this study and the intrinsic DNA structure properties. In one group of simulation, ten DNA sequences used. They are $\begin{matrix}5'\text{-GTACN}\mathbf{CG}\text{BGTAC-}3' \\ 3'\text{-CATGB}\mathbf{GC}\text{NCATG-}5'\end{matrix}$, which contain all the 16 combinations of N_5CGN_3 in the study of the de novo methylation. In these sequences, CpG was designed to be in the middle of the double helix to minimize the effect of the finite chain length. In another group ten $\begin{matrix}5'\text{-GTACN5}\mathbf{5mCG}\text{N3GTAC-}3' \\ 3'\text{-CATGN3}\mathbf{G5mC}\text{N5CATG-}5'\end{matrix}$ sequences are used, which all contain two methylated CpG sites. In these calculations, except for the four middle N_5XGN_3 bases, the DNAs are designed to have the identical palindromic sequence to avoid other flanking sequence effects.

All simulations were conducted using AMBER suite of programs [1, 2]. In each system, a canonical B form DNA was chosen as the initial structure. The DNA was then immersed into a cubic box containing water (SPC/E model [3]) and sodium ions as counterions. The water layer surrounding the DNA is 12 Å thick. Nucleic acid parameters were taken from the AMBER ff10 parameter set [4]. The force field of 5-methylcytosine was obtained from the work of SantaLucia [5]. For each system, the simulation procedure included the energy minimization, heating-up and a production equilibrium simulation using the NPT ensemble. The system was first prepared through 500 steps of steepest descent minimization and a following 500 steps of conjugate gradient minimization with DNA being fixed using harmonic restraints. Then the restraints on DNA were released and the system was further minimized using 1000 steps of steepest descent and then 1500 steps of conjugate gradient minimization. For further relaxation, the systems were heated to 300K and then equilibrated**.** Production runs were performed and the temperature of all systems was maintained at 300K using the Langevin dynamics with a friction coefficient of $5ps^{-1}$. The simulation times are all 200ns, which give a total simulation time of $4\mu S$. The pressure of the system was adjusted to 1 atm by Berendsen weak-coupling algorithm with a relaxation time constant of 2.0 ps [6]. In all simulations, the SHAKE algorithm was used to restraint all covalent bonds involving hydrogen atoms [7]. All dynamic runs employed an integral time step of 2 fs. Periodic boundary conditions were used and the particle-mesh Ewald(PME) with a direct space cutoff of 10.0Å was utilized to treat long-range electrostatic interactions [8].

Supporting reference

1. Pearlman DA, Case DA, Caldwell JW, Ross WS, Cheatham TE, Debolt S, et al. Amber, a package of computer-programs for applying molecular mechanics, normal-mode analysis, molecular-dynamics and free-energy calculations to simulate the structural and energetic properties of molecules. Comput Phys Commun. 1995;91(1-3):1-41.

2. Case DA, Cheatham TE, Darden T, Gohlke H, Luo R, Merz KM, et al. The Amber biomolecular simulation programs. J Comput Chem. 2005;26(16):1668-88.

3. Berendsen HJC, Grigera JR, Straatsma TP. The missing term in effective pair potentials. J Phys Chem. 1987;91(24):6269-71.

4. Perez A, Marchan I, Svozil D, Sponer J, Cheatham TE, Laughton CA, et al. Refinenement of the AMBER force field for nucleic acids: Improving the description of alpha/gamma conformers. Biophys J. 2007;92(11):3817-29.

5. Aduri R, Psciuk BT, Saro P, Taniga H, Schlegel HB, SantaLucia J. AMBER force field parameters for the naturally occurring modified nucleosides in RNA. J Chem Theory Comput. 2007;3(4):1464-75.

6. Berendsen HJC, Postma JPM, Vangunsteren WF, Dinola A, Haak JR. Molecular-dynamics with coupling to an external bath. J Chem Phys. 1984;81(8):3684-90.

7. Ryckaert JP, Ciccotti G, Berendsen HJC. Numerical-integration of cartesian equations of motion of a system with constraints - molecular-dynamics of n-alkanes. J Comput Phys. 1977;23(3):327-41.

8. Darden T, York D, Pedersen L. Particle mesh ewald - an n.log(n) method for ewald sums in large systems. J Chem Phys. 1993;98(12):10089-92.
