# Supplementary material for: The sequence preference of DNA methylation variation in mammalians
Source: PLoS One. 2017 Oct 18;12(10):e0186559. doi: 10.1371/journal.pone.0186559 (PMC5646869; doi:10.1371/journal.pone.0186559)
Supplement: S1 Fig — (PDF) [file pone.0186559.s002.pdf]

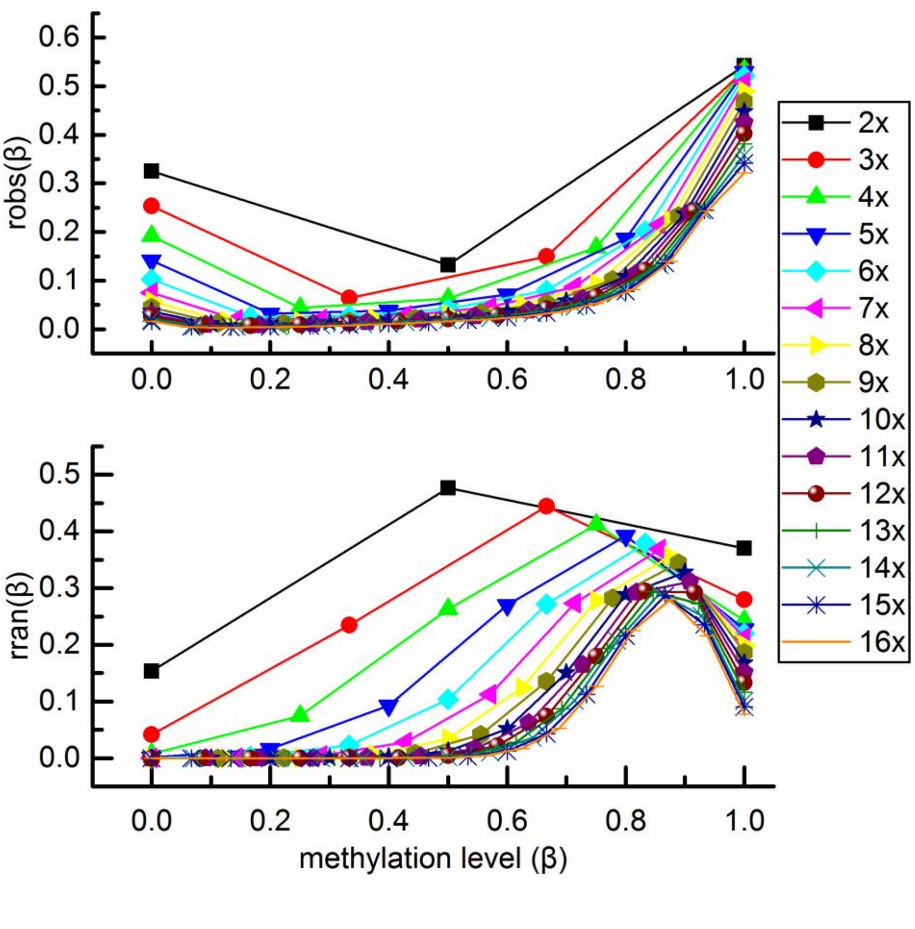

Figure S1. The observed and random distribution of CpG methylation in different sequencing depths. The observed methylation level distribution is a bimodal distribution no matter of the sequencing depth while the random distribution is unimodal as we assume the methylation frequency n obeys a binomial distribution (Eq 3). The final random distribution is the combination of the random distribution of all sequencing depth (Fig 1A).
