# Supplementary material for: The sequence preference of DNA methylation variation in mammalians
Source: PLoS One. 2017 Oct 18;12(10):e0186559. doi: 10.1371/journal.pone.0186559 (PMC5646869; doi:10.1371/journal.pone.0186559)
Supplement: S2 Fig — (PDF) [file pone.0186559.s003.pdf]

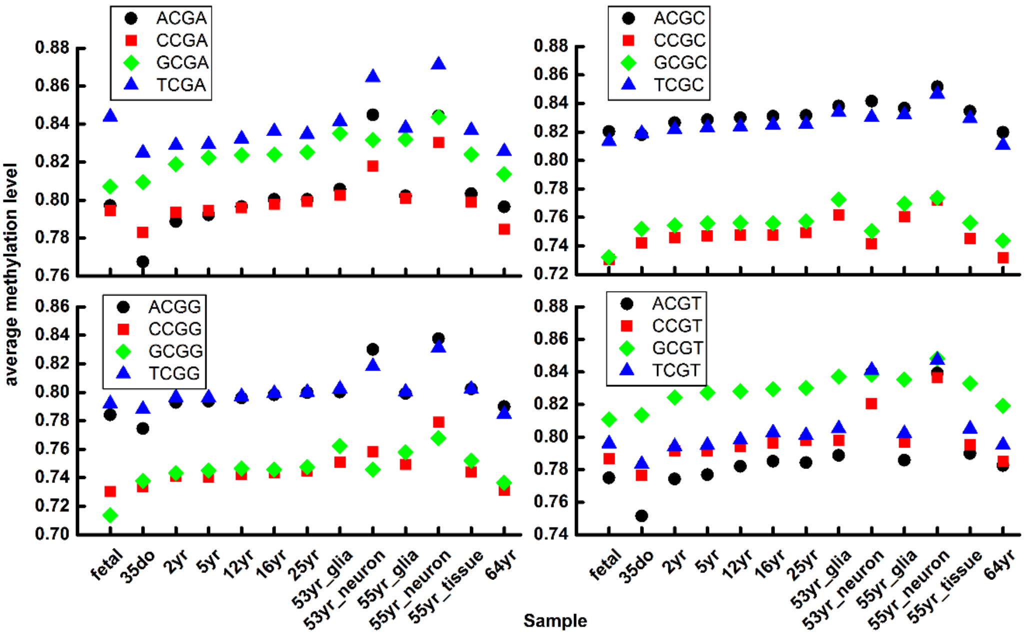

Figure S1. Average methylation level of N_5CGA (upper left), N_5CGC (upper right), N_5CGG (lower left) and N_5CGT (lower right) in human brain cells. The average methylation level of $ACGN_3$, $CCGN_3$, $GCGN_3$ and $TCGN_3$ are represented as black circle, red square, green diamond and blue triangle, respectively.
