# Supplementary material for: The sequence preference of DNA methylation variation in mammalians
Source: PLoS One. 2017 Oct 18;12(10):e0186559. doi: 10.1371/journal.pone.0186559 (PMC5646869; doi:10.1371/journal.pone.0186559)
Supplement: S3 Fig — (PDF) [file pone.0186559.s004.pdf]

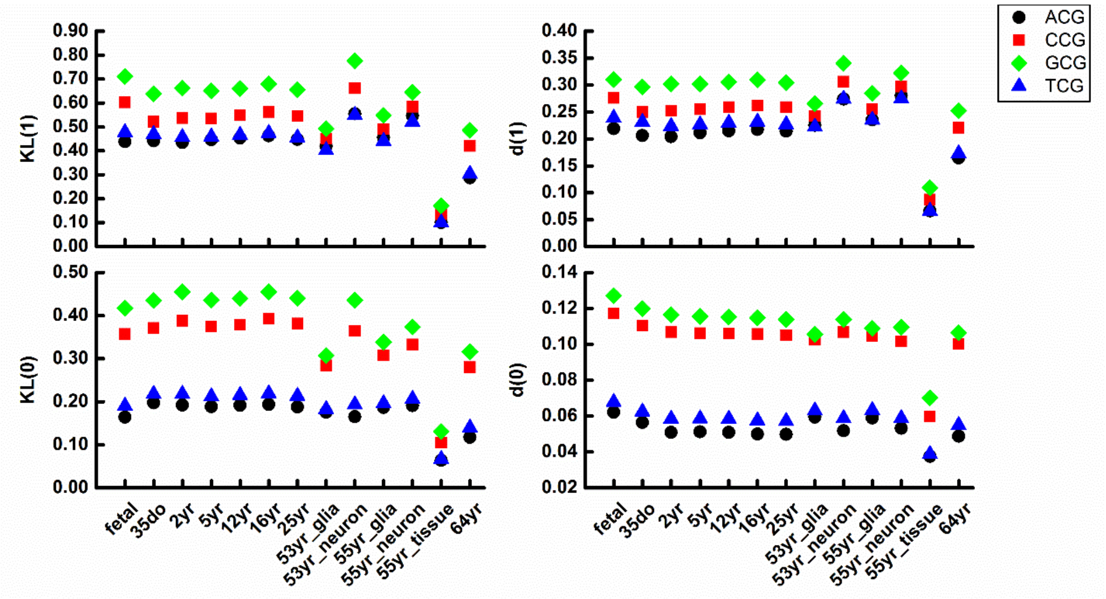

Figure S1. Comparison between the d(0), KL(0) and d(1), KL(1) in the chromosome 1 of human brain samples. Both the direct difference and the relative entropy are good descriptor of the trend.
