# Supplementary material for: The sequence preference of DNA methylation variation in mammalians
Source: PLoS One. 2017 Oct 18;12(10):e0186559. doi: 10.1371/journal.pone.0186559 (PMC5646869; doi:10.1371/journal.pone.0186559)
Supplement: S5 Fig — (PDF) [file pone.0186559.s006.pdf]

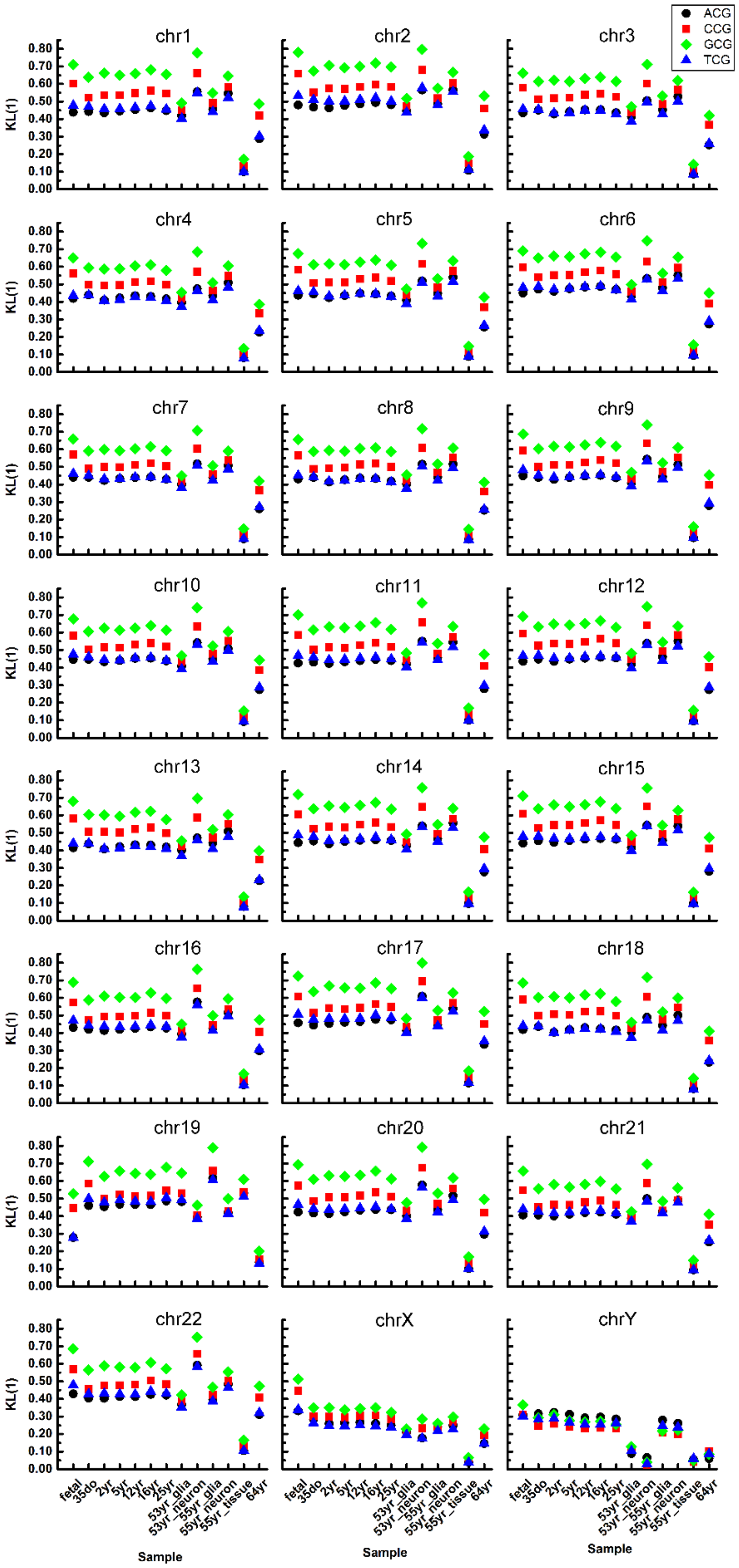

Figure S5. The demethylation variation of trinucleotides in all chromosomes in human brain samples. The demethylation variation of GCG and CGG is lower than that of ACG and TCG in all chromosomes.
