# Supplementary material for: The sequence preference of DNA methylation variation in mammalians
Source: PLoS One. 2017 Oct 18;12(10):e0186559. doi: 10.1371/journal.pone.0186559 (PMC5646869; doi:10.1371/journal.pone.0186559)
Supplement: S6 Fig — (PDF) [file pone.0186559.s007.pdf]

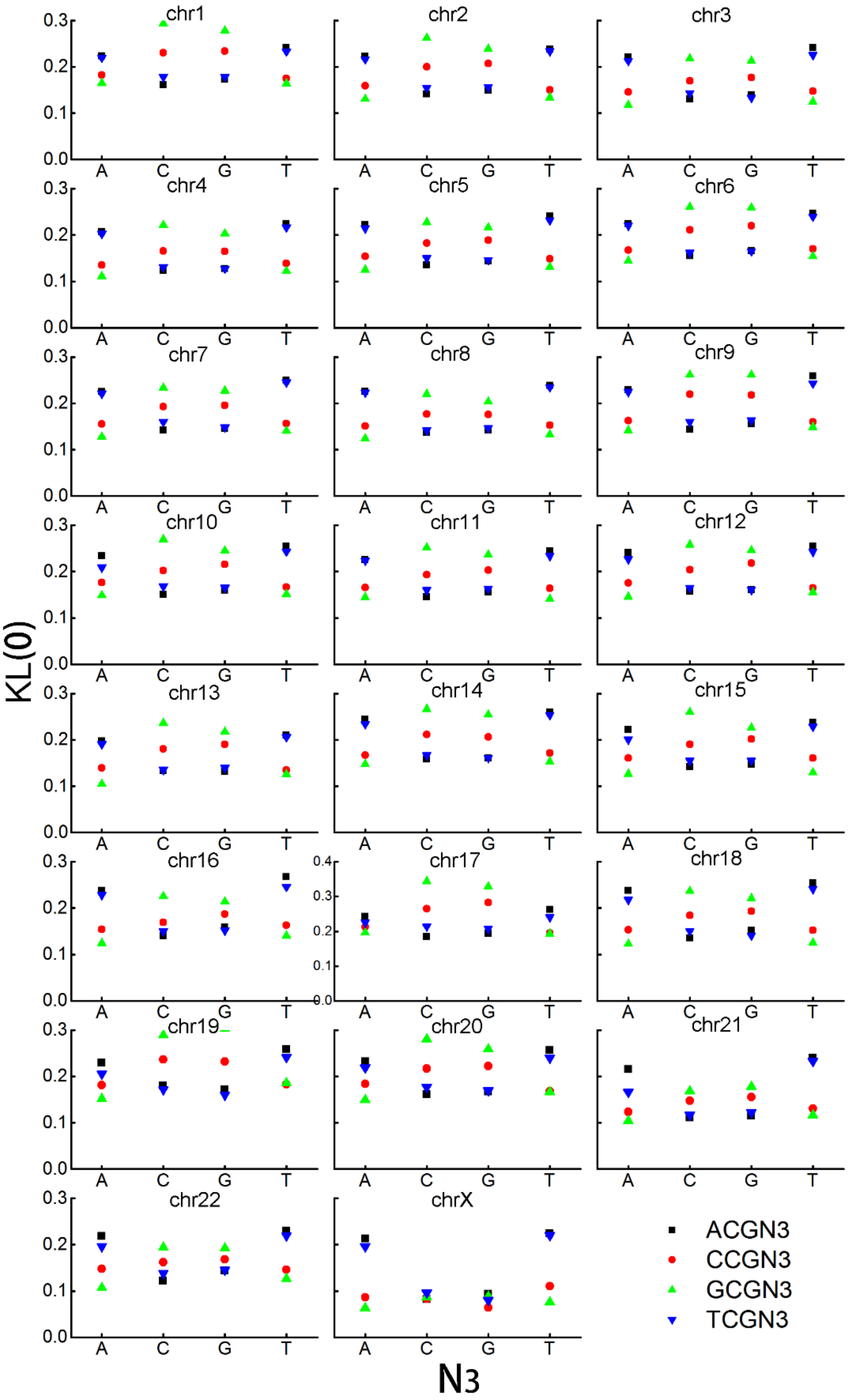

Figure S6. The methylation variation of tetranucleotide in PMDs of all chromosomes in IMR90 cell line.
