# Supplementary material for: The sequence preference of DNA methylation variation in mammalians
Source: PLoS One. 2017 Oct 18;12(10):e0186559. doi: 10.1371/journal.pone.0186559 (PMC5646869; doi:10.1371/journal.pone.0186559)
Supplement: S8 Fig — (PDF) [file pone.0186559.s009.pdf]

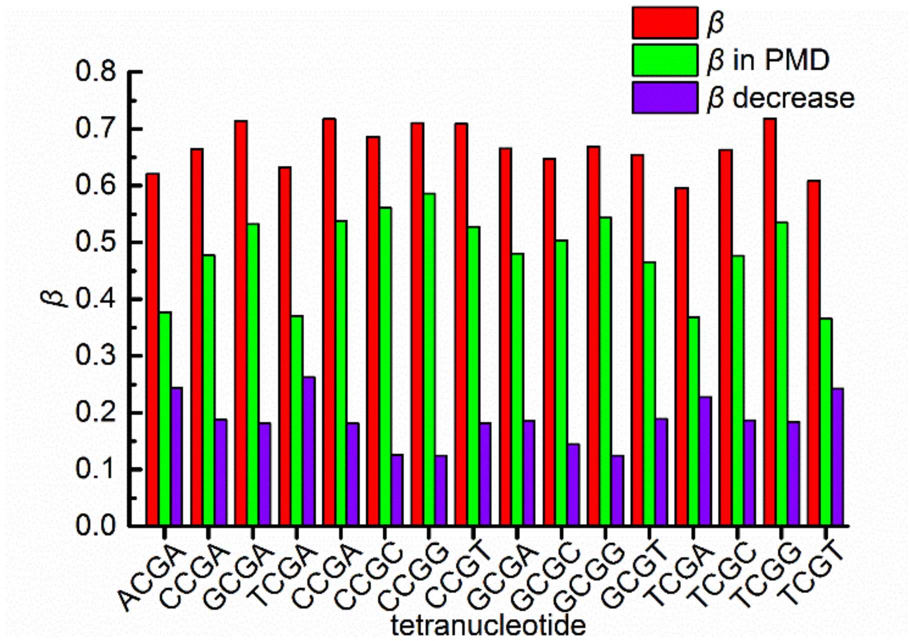

Figure S8. The average methylation level of the tetranucleotides of chromosome 1 in IMR90 cell lines. The red bar is the average methylation level of all N_5CGN_3 tetranucleotides in chromosome 1 and the green bars is the average methylation level of N_5CGN_3 tetranucleotides located in PMDs in chromosome 1. The purple bars are the difference between the red bar and the green bar. The GCGC, GCGG, CCGC and GCGG have minor methylation level change.
