# Supplementary material for: The sequence preference of DNA methylation variation in mammalians
Source: PLoS One. 2017 Oct 18;12(10):e0186559. doi: 10.1371/journal.pone.0186559 (PMC5646869; doi:10.1371/journal.pone.0186559)
Supplement: S9 Fig — The distributions of SASA of O2 for (A) N5CGA, (B) N5CGC, (C) N5CGG and (D) N5CGT. (PDF) [file pone.0186559.s010.pdf]

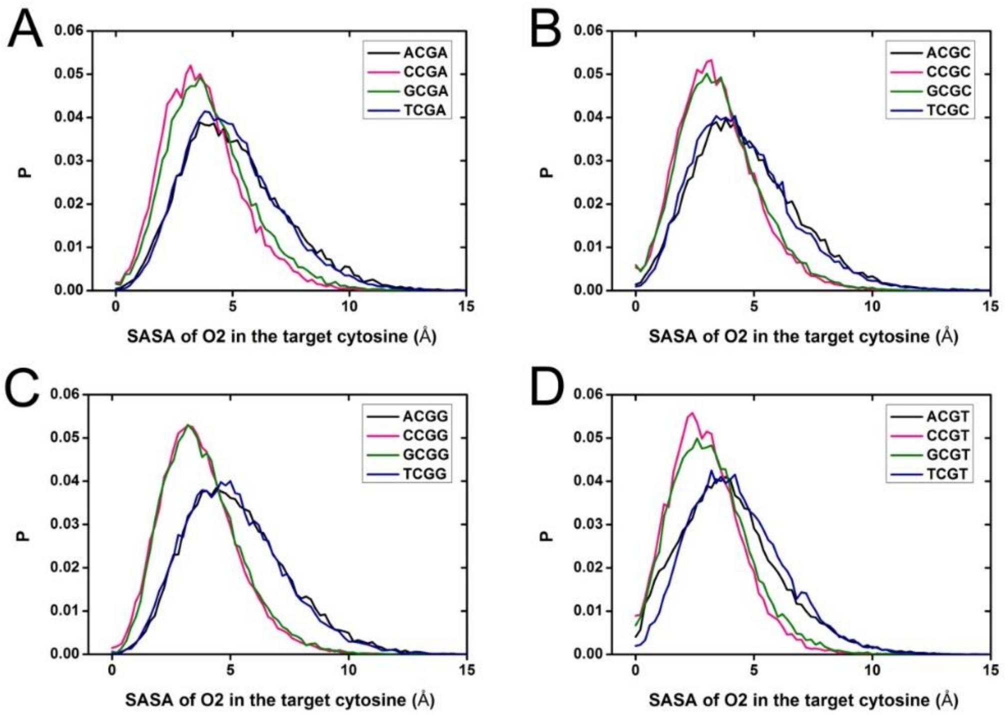

Figure S9. The distributions of SASA of O2 atom for (A) N$_5$CGA, (B) N$_5$CGC, (C) N$_5$CGG and (D) N$_5$CGT. The distributions of ACGN$_3$, CCGN$_3$, GCGN$_3$ and TCGN$_3$ are in black, red, green and blue, respectively. N$_5$, N$_3$=A, C, G or T.
