# Supplementary material for: The sequence preference of DNA methylation variation in mammalians
Source: PLoS One. 2017 Oct 18;12(10):e0186559. doi: 10.1371/journal.pone.0186559 (PMC5646869; doi:10.1371/journal.pone.0186559)
Supplement: S10 Fig — The distributions of SASA of O2 for (A) N5mCGA, (B) N5mCGC, (C) N5mGG and (D) N5mCGT. (PDF) [file pone.0186559.s011.pdf]

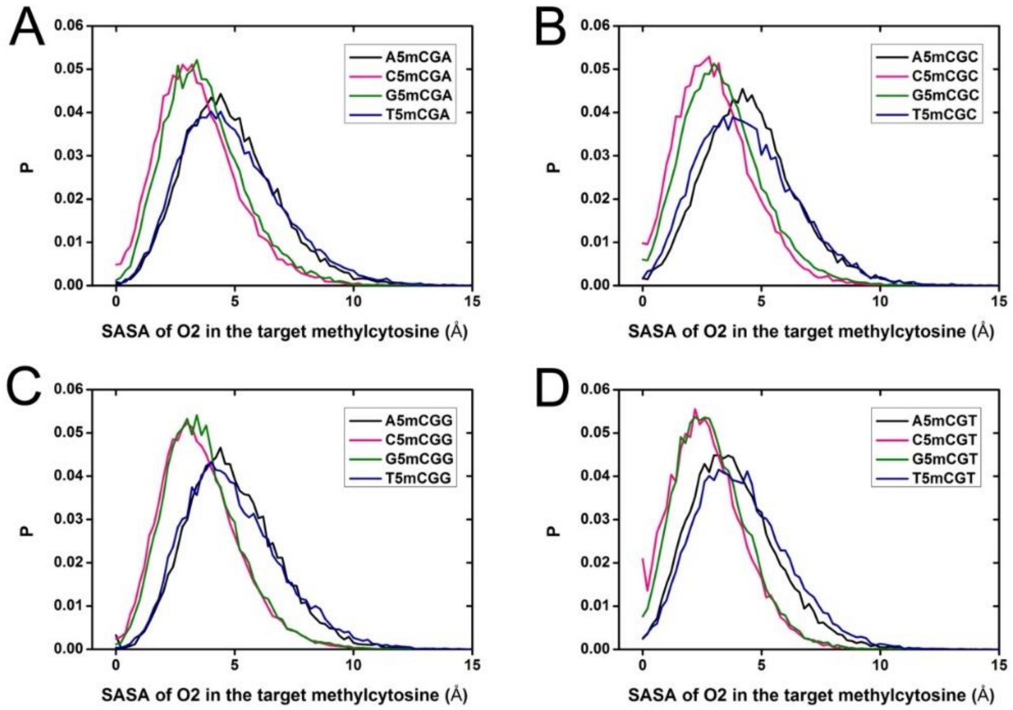

Figure S10. The distributions of SASA of O2 atom for (A) N_5mCGA, (B) N_5mCGC, (C) N_5mGG and (D) N_5mCGT. The distributions of AmCGN_3, CmCGN_3, GmCGN_3 and TmCGN_3 are in black, red, green and blue, respectively. N_5, N_3=A, C, G or T.
