# Supplementary material for: The sequence preference of DNA methylation variation in mammalians
Source: PLoS One. 2017 Oct 18;12(10):e0186559. doi: 10.1371/journal.pone.0186559 (PMC5646869; doi:10.1371/journal.pone.0186559)
Supplement: S11 Fig — The distribution of bending magnitudes of CpG sites relate to (A) N5CGA, (B) N5CGC, (C) N5CGG and (D) N5CGT. (PDF) [file pone.0186559.s012.pdf]

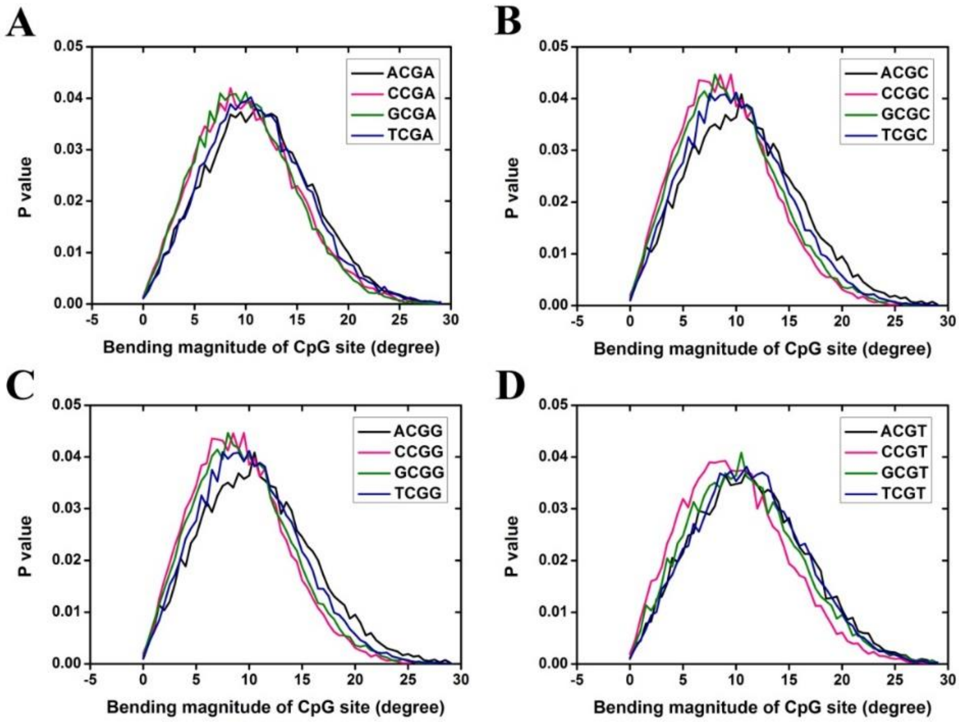


**Figure S11. The distribution of bending magnitudes of CpG sites relate to (A) $N_5$CGA, (B) $N_5$CGC, (C) $N_5$CGG and (D) $N_5$CGT.** The distributions of ACG$N_3$, CCG$N_3$, GCG$N_3$ and TCG$N_3$ are in black, red, green and blue, respectively. $N_5$, $N_3$=A, C, G or T.
