# Supplementary material for: The sequence preference of DNA methylation variation in mammalians
Source: PLoS One. 2017 Oct 18;12(10):e0186559. doi: 10.1371/journal.pone.0186559 (PMC5646869; doi:10.1371/journal.pone.0186559)
Supplement: S12 Fig — The distribution of bending magnitudes of 5mCpG sites relate to (A) N5mCGA, (B) N5mCGC, (C) N55mCGG and (D) N5mCGT. (PDF) [file pone.0186559.s013.pdf]

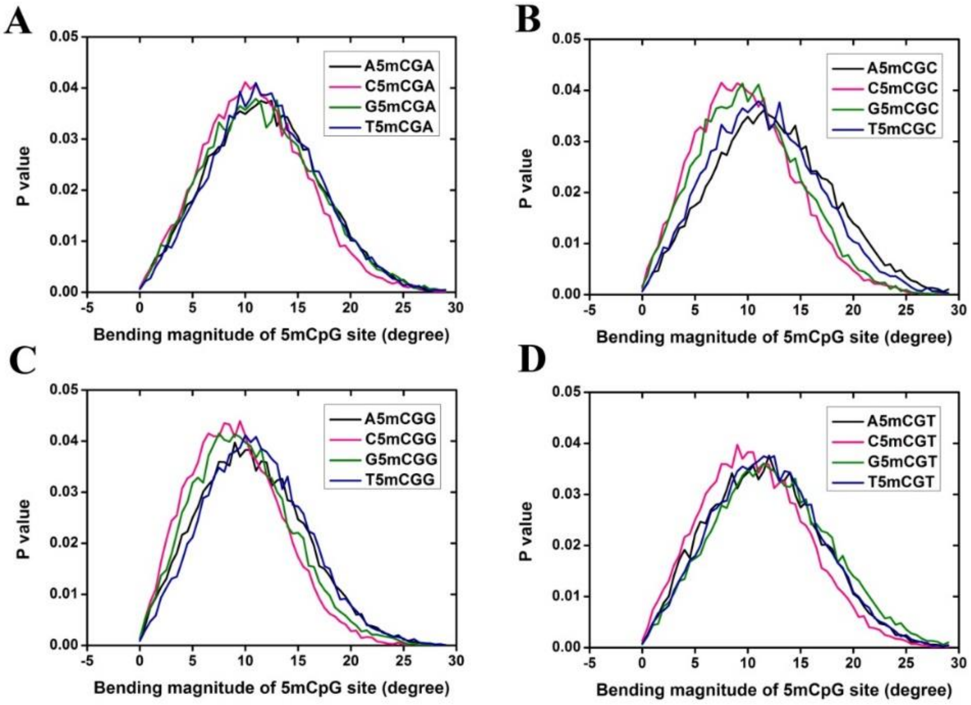

Figure S12. The distribution of bending magnitudes of mCpG sites relate to (A) N_5mCGA, (B) N_5mCGC, (C) $N_5$5mCGG and (D) N_5mCGT. The distributions of AmCGN_3, CmCGN_3, GmCGN_3 and TmCGN_3 are in black, red, green and blue, respectively. N_5, N_3=A, C, G or T. AmCGN_3 and TmCGN_3 have higher average bending magnitudes than GmCGN_3 and CmCGN_3, except that GmCGT has the highest value in N_5mCGT.
