# Supplementary material for: The sequence preference of DNA methylation variation in mammalians
Source: PLoS One. 2017 Oct 18;12(10):e0186559. doi: 10.1371/journal.pone.0186559 (PMC5646869; doi:10.1371/journal.pone.0186559)
Supplement: S1 Table — (PDF) [file pone.0186559.s014.pdf]

Table S1 The detailed information of human brain samples

symbol	brain region	cell type	gender	age
fetal	cerebral cortex	tissue	M	20 week
35do	middle frontal gyrus	tissue	M	35 day
2yr	middle frontal gyrus	tissue	M	2 year
5yr	middle frontal gyrus	tissue	M	5 year
12yr	middle frontal gyrus	tissue	M	12 year
16yr	middle frontal gyrus	tissue	M	16 year
25yr	middle frontal gyrus	tissue	M	25 year
53yr_neuron	dorsal prefrontal cortex	neurons	F	53 year
53yr_glia	dorsal prefrontal cortex	glia	F	53 year
55yr_tissue	dorsal prefrontal cortex	tissue	M	55 year
55yr_neuron	dorsal prefrontal cortex	neurons	M	55 year
55yr_glia	dorsal prefrontal cortex	glia	M	55 year
64yr	frontal cortex	grey matter	F	64 year

*URL: http://neomorph.salk.edu/brain_methylomes/
