# Supplementary material for: The sequence preference of DNA methylation variation in mammalians
Source: PLoS One. 2017 Oct 18;12(10):e0186559. doi: 10.1371/journal.pone.0186559 (PMC5646869; doi:10.1371/journal.pone.0186559)
Supplement: S2 Table — (PDF) [file pone.0186559.s015.pdf]

Table S2 The detailed information of mouse brain samples

symbol	brain region	cell type	gender	age
fetal	frontal cortex	tissue	-	e13
1w	frontal cortex	tissue	M	1 week
2w	frontal cortex	tissue	M	2 week
4w	frontal cortex	tissue	M	4 week
6w	frontal cortex	tissue	M	6 week
10w	frontal cortex	tissue	M	10 week
22mo	frontal cortex	tissue	M	22 month
7w_neuron	frontal cortex	neurons	M	7 week
7w_glia	frontal cortex	glia	M	7 week
6w_neuron	frontal cortex	neurons	F	6 week
6w_glia	frontal cortex	glia	F	6 week
12mo_neuron	frontal cortex	neurons	F	12 month
12mo_glia	frontal cortex	glia	F	12 month

*URL: http://neomorph.salk.edu/brain_methylomes/
