# Supplementary material for: The sequence preference of DNA methylation variation in mammalians
Source: PLoS One. 2017 Oct 18;12(10):e0186559. doi: 10.1371/journal.pone.0186559 (PMC5646869; doi:10.1371/journal.pone.0186559)
Supplement: S3 Table — (PDF) [file pone.0186559.s016.pdf]

Table S3 The detailed information of human ESCs and iPSCs samples

name	symbol	cell type
ADS	ads	adipose derived stem cells
ADS-adipose	ads_adipose	adipocytes derived from the ADS cells
IMR90	imr90	fetal lung fibroblasts
ff	ff	foreskin fibroblasts
H1	h1	embryonic stem cells
Hues6(human embryonic stem cells 6)	hues6	embryonic stem cells
H9	h9	embryonic stem cells
ADS-iPSC	ads_ipsc	induced pluripotent stem cells
IMR90-iPSC (fetal lung fibroblast derived)	imr90_ipsc	induced pluripotent stem cells
FF iPSC 6.9 H (foreskin fibroblast derived)	ff_ipsc_6_9	induced pluripotent stem cells
FF iPSC 19.7 F (foreskin fibroblast derived)	ff_ipsc_19_7	induced pluripotent stem cells
FF iPSC 19.11 B (foreskin fibroblast derived)	ff_ipsc_19_11	induced pluripotent stem cells

*URL: http://neomorph.salk.edu/ips_methylomes

*For hues6, the URLis http://neomorph.salk.edu/brain_methylomes/
