# Supplementary material for: The sequence preference of DNA methylation variation in mammalians
Source: PLoS One. 2017 Oct 18;12(10):e0186559. doi: 10.1371/journal.pone.0186559 (PMC5646869; doi:10.1371/journal.pone.0186559)
Supplement: S4 Table — (PDF) [file pone.0186559.s017.pdf]

Table S4 The detailed information of human somatic cell samples

name	symbol	gender	Methylome GEO Accessions
adrenal	adrenal	F	GSM1282354
aorta	aorta	F	GSM1282355
esophagus	esophagus	F	GSM1282356
gastric	gastric	F	GSM1282358
lung	lung	F	GSM983647,GSM1010985,GSM1059422
ovary	ovary	F	GSM1010980,GSM1120323,GSM1059423
pancreas	pancreas	F	GSM1282359
spleen	spleen	F	GSM1282362

*URL: http://neomorph.salk.edu/human_tissue_methylomes.html
