# Supplementary material for: The sequence preference of DNA methylation variation in mammalians
Source: PLoS One. 2017 Oct 18;12(10):e0186559. doi: 10.1371/journal.pone.0186559 (PMC5646869; doi:10.1371/journal.pone.0186559)
Supplement: S5 Table — (PDF) [file pone.0186559.s018.pdf]

Table S5 The detailed information of human PGC samples

name	symbol	gender	developmental stage
PGC_7W_embryo1	7w_mpgc1	M	7 week gestation
PGC_7W_embryo2	7w_mpgc2	M	7 week gestation
PGC_10W_embryo1	10w_mpgc	M	10 week gestation
PGC_10W_embryo1_rep1	10w_fpgc1	F	10 week gestation
PGC_10W_embryo1_rep2	10w_fpgc2	F	10 week gestation
PGC_11W_embryo1_rep1	11w_mpgc1	M	11 week gestation
PGC_11W_embryo1_rep2	11w_mpgc2	M	11 week gestation
PGC_11W_embryo1	11w_fpgc	F	11 week gestation
PGC_13W_embryo1	13w_mpgc	M	13 week gestation
PGC_17W_embryo1	17w_fpgc	F	17 week gestation
PGC_19W_embryo1	19w_mpgc1	M	19 week gestation
PGC_19W_embryo2	19w_mpgc2	M	19 week gestation

*Accession number : GSE63818
