# Supplementary material for: The sequence preference of DNA methylation variation in mammalians
Source: PLoS One. 2017 Oct 18;12(10):e0186559. doi: 10.1371/journal.pone.0186559 (PMC5646869; doi:10.1371/journal.pone.0186559)
Supplement: S6 Table — (PDF) [file pone.0186559.s019.pdf]

Table S6 The detailed information of human gonadal somatic (SOMA) samples

name	symbol	gender	developmental stage
Brain_5W_embryo1	5w_brain	M	5 week gestation
Heart_5W_embryo1	5w_heart	M	5 week gestation
Soma_7W_embryo1	7w_msoma1	M	7 week gestation
Soma_7W_embryo2	7w_msoma2	M	7 week gestation
Soma_10W_embryo1	10w_msoma	M	10 week gestation
Soma_11W_embryo1	11w_msoma	M	11 week gestation
Soma_17W_embryo1	17w_fsoma	F	17 week gestation
Soma_19W_embryo1	19w_msoma	M	19 week gestation

*Accession number : GSE63818
